# Comparison of neuronal GAL4 drivers along with the AGES (auxin-inducible gene expression system) and TARGET (temporal and regional gene expression targeting) systems for fine tuning of neuronal gene expression in *Drosophila*

**DOI:** 10.17912/micropub.biology.000885

**Published:** 2023-06-16

**Authors:** Hannah R Hawley, Celestine J Roberts, Helen L Fitzsimons

**Affiliations:** 1 School of Natural Sciences, Massey University, Palmerston North, Manawatu-Wanganui, New Zealand

## Abstract

Spatial and temporal control of gene expression in *Drosophila* is essential in elucidating gene function. Spatial control is facilitated by the UAS/GAL4 system, and this can be coupled with additional adaptations for precise temporal control and fine tuning of gene expression levels. Here we directly compare the level of pan-neuronal transgene expression governed by nSyb-GAL4 and elav-GAL4, as well as mushroom body-specific expression alongside OK107-GAL4. We also compare the temporal modulation of gene expression in neurons with the auxin-inducible gene expression system (AGES) and temporal and regional gene expression targeting (TARGET) systems.

**Figure 1. Comparison of levels and patterns of expression of neuronal drivers in the brain and fine-tuning of expression levels using AGES and TARGET systems:**
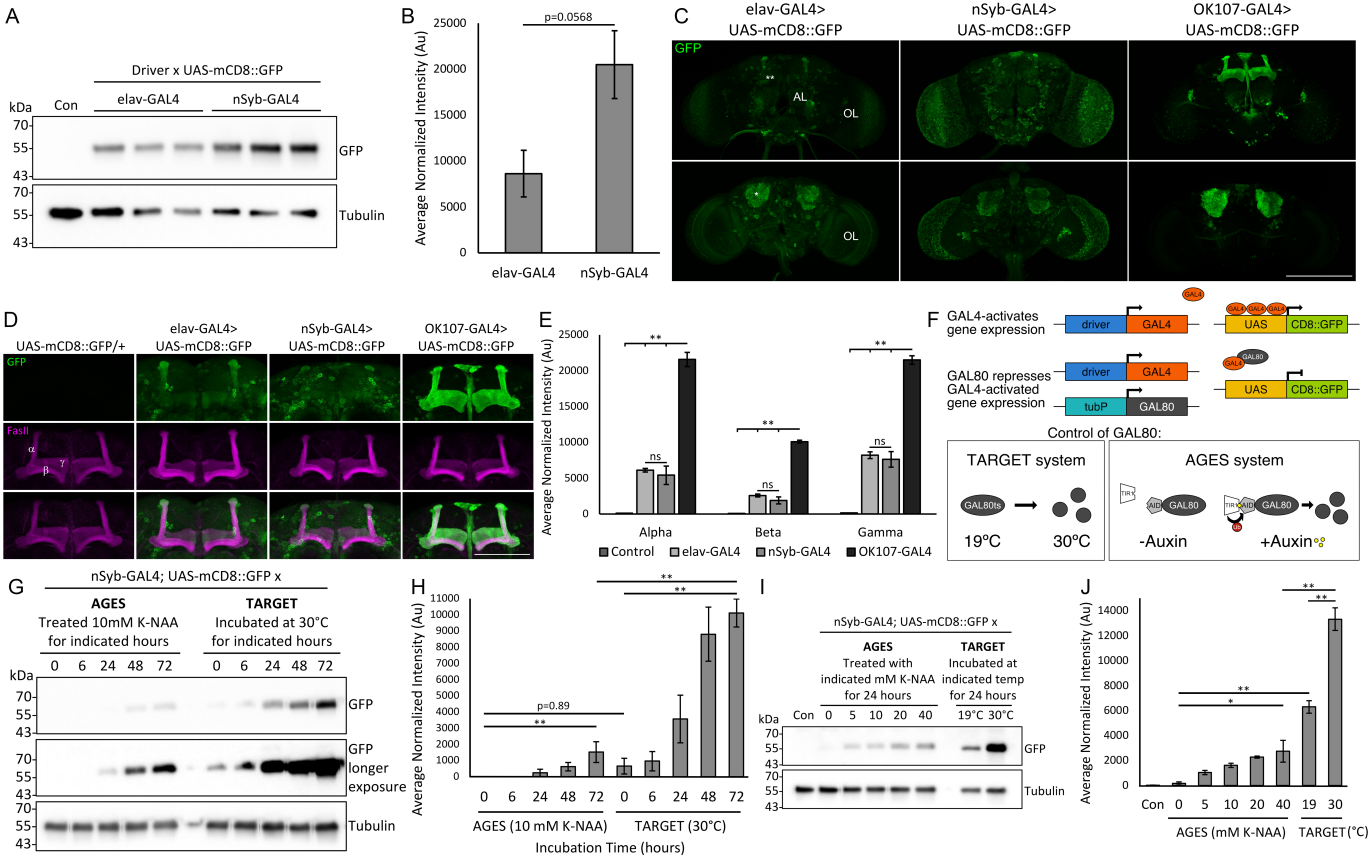
A. Western blot of whole cell lysates generated from heads of flies expressing mCD8::GFP under the control of either elav-GAL4 or nSyb-GAL4 to compare relative levels of expression. mCD8::GFP is observed at the expected size of ~55 kDa. The blot was stripped and reprobed with anti-tubulin as a loading control. Samples processed from three independent crosses were loaded. Con, wild-type Canton S. B. Quantification of A. Mean mCD8::GFP band intensity (±SEM) normalized to tubulin is shown. mCD8::GFP intensity is reduced when driven by elav-GAL4 compared to nSyb-GAL4, but this does not quite reach statistical significance (Students t-test, t(4)=2.6533, p=0.0568). C. Brains of flies expressing mCD8::GFP under the control of elav-GAL4, nSyb-GAL4 and OK107-GAL4 were stained with anti-GFP to assess distribution. All images are maximum projections through the anterior (top row) or posterior (bottom row) of the brain. AL, antennal lobe; OL, optic lobe; ** gamma lobe of the mushroom body; * calyx. Scale bar 100 µm. D. Maximum projections through the anterior of the brain of flies expressing mCD8::GFP (green) under the control of elav-GAL4, nSyb-GAL4 and OK107-GAL4. Brains are counterstained with anti-FasII (magenta) to label the alpha (α), beta (β) and gamma lobes (γ). Scale bar 200 µm. E. Quantification of D. Lobes of the mushroom body (alpha, beta, and gamma) were traced on a single optical section (FasII channel), then the intensity of mCD8::GFP was measured and normalized to FasII. mCD8::GFP signal outside of the alpha, beta, and gamma lobe outlines provided by FasII counterstaining was not recorded. OK107-GAL4>mCD8::GFP intensity is significantly increased compared to both elav-GAL4>mCD8::GFP and nSyb-GAL4>mCD8::GFP in each of the alpha, beta, and gamma lobes (ANOVA, alpha F _(3,28)_ =128, p<0.001; beta F _(3,28)_ =251, p<0.001; gamma F _(3,28)_ =182, p<0.001; post-hoc Tukey’s HSD, **p<0.01). There is no statistically significant difference in normalized mCD8::GFP intensity between elav-GAL4 and nSyb-GAL4 in any of the lobes. Mean ±SEM is shown, n=8 lobes/genotype. F. Schematic diagram depicting regulation of GAL4 activity mediated by the temporal and regional gene expression targeting (TARGET) and auxin-inducible gene expression system (AGES) systems. GAL4 is a transcriptional activator which binds at upstream activating sequences (UAS) to facilitate transgene expression. GAL4 expression is mediated in a tissue-specific manner by upstream regulatory elements (driver). Both systems utilise GAL80, a repressor of GAL4, to prevent GAL4-induced gene expression. GAL80 is under the control of the tubulin promoter, resulting in ubiquitous expression. In the TARGET system, flies harbour a temperature sensitive mutant of GAL80 (GAL80ts) which is inactivated at temperatures above 30°C, resulting in GAL4-mediated transgene expression. In the AGES system, GAL80 is fused to an auxin-inducible degron (AID) tag, which in the presence of auxin binds the F-box protein TIR. TIR then ubiquitinates AID, thus promoting degradation of GAL80. G. Western blot comparing temporal induction profiles of mCD8::GFP mediated by nSyb-GAL4 under the control of the AGES and TARGET systems. Gene expression was induced by either transfer of adult flies to food containing 10 mM K-NAA (AGES) or transfer from 19°C to 30°C (TARGET) for the indicated number of hours. Whole-cell lysates were then generated from heads and blots probed with anti-GFP, followed by anti-tubulin. AGES, UAS-tubP-TIR1-2A-GAL80.AID. TARGET, UAS-tubP-GAL80ts. Con, wild-type Canton S. The experiment was performed in triplicate and a representative blot is shown. H. Quantification of G. Mean mCD8::GFP band intensity (±SEM) normalized to tubulin is shown. mCD8::GFP intensity at 0 hours for TARGET is increased compared to AGES indicating incomplete repression, however this is not statistically significant (ANOVA, F _(9.20)_ =20.6, p<0.001, post-hoc Tukey’s HSD p=0.89). Maximum mCD8::GFP expression is achieved at 72 hours for each system (**p<0.01 compared to 0 hours), and at each time point TARGET is significantly increased compared to AGES (**p<0.01). I. Western blot illustrating dose-dependent induction of mCD8::GFP expression mediated by GAL4 in the presence of AGES at the 24 hour time point. Adult flies were transferred to food containing the indicated concentration of K-NAA for 24 hours. Whole-cell lysates were then generated from heads and blots probed with anti-GFP, followed by anti-tubulin. AGES, UAS-tubP-TIR1-2A-GAL80.AID. TARGET, UAS-tubP-GAL80ts. Con, control, is wild-type (Canton S). The experiment was performed in triplicate and a representative blot is shown. J. Quantification of I. Mean mCD8::GFP band intensity (±SEM) normalized to tubulin is shown. mCD8::GFP expression increased with increasing dose of K-NAA, but did not reach the level of induction achieved by the TARGET system (30°C) at 24 hours (ANOVA, F _(7,16)_ =83.5, p<0.001, post-hoc Tukey’s HSD *p<0.05, **p<0.001).

## Description

Precise manipulation of transgene expression with respect to both space and time is a cornerstone of discovery in *Drosophila melanogaster* , and is widely facilitated by the UAS/GAL4 system coupled with various adaptations. This is a bipartite system that governs spatial control; tissue-specific promoters drive expression of the yeast transcriptional transactivator GAL4 which bind to upstream activating sequences (UAS) to induce transgene expression a tissue-specific pattern (Brand & Perrimon, 1993) ( Figure 1F ). Given the vast resources already available for this system, compatible adaptations for addition of temporal control are most desirable. GAL80 is a repressor of GAL4 and thus prevents activation of UAS-based gene expression (Lee & Luo, 1999) . Regulation of GAL80 stability is used to facilitate temporal control and has been achieved by introduction of a temperature sensitive mutant of GAL80 (GAL80ts) in the TARGET (temporal and regional gene expression targeting) system (McGuire *et al.* , 2004). More recently, the auxin-inducible gene expression system (AGES) has been developed, which regulates GAL80 activity via drug-dependent degradation (McClure *et al.* , 2022). GAL80 is fused to an auxin-inducible degron (AID), which in the presence of auxin binds TIR, an F-box protein that recruits Skp1 and Cullin to form an E3 ligase complex. This complex then ubiquitinates AID resulting in auxin-dependent degradation of the AID-GAL80 fusion, and derepression of GAL4. While these systems are typically used to restrict transgene expression to a specific developmental stage, they also facilitate tailoring of the level of transgene expression; we previously demonstrated that presence of GAL80ts allows for linear increases in transgene expression in a temperature-sensitive manner (Schwartz *et al.* , 2016).

Choice of both driver and regulatory systems to facilitate careful control of gene expression is essential in successful experimental design. Two commonly used pan-neuronal drivers include elav-GAL4 (Robinow & White, 1988) and nSyb-GAL4 (Riabinina *et al.* , 2015). Data on their expression patterns in the adult brain is available (Armstrong *et al.* , 2011; Weaver *et al.* , 2020) however we have not found direct comparisons of levels of expression mediated by each of these in the literature, which is essential in interpretation of dose-dependent phenotypes. Here we provide a direct comparison of expression levels of these drivers in whole brain. Due to our interest in development and function of the mushroom body (Freymuth & Fitzsimons, 2017; Main *et al.* , 2021; Palmer & Fitzsimons, 2023; Schwartz *et al.* , 2023) we also compared the level of expression driven by nSyb-GAL4 and elav-GAL4 to that of the mushroom body driver OK107-GAL4 (Connolly *et al.* , 1996). Finally, we characterized and compared the AGES and TARGET systems in their ability to provide temporal, tunable control of nSyb-GAL4-mediated gene expression in a drug and temperature-dependent manner, respectively.

We first examined expression levels of mCD8::GFP, a membrane targeted GFP marker (Lee & Luo, 1999) , mediated by the two neuronal drivers elav-GAL4 and nSyb-GAL4 in whole head lysates ( Figure 1A ). Expression of mCD8::GFP was 2.4 times higher when driven by nSyb-GAL4 compared to elav-GAL4 ( Figure 1B ). Immunohistochemistry on brains expressing mCD8::GFP with each driver revealed different expression patterns ( Figure 1C ), which were consistent with their previously described distributions (Armstrong *et al.* , 2011; Fitzsimons & Scott, 2011; Weaver *et al.* , 2020). nSyb-GAL4 expression was higher and more widespread throughout the optic lobes compared to elav-GAL4, which appeared higher in cell bodies of the mushroom body at the posterior of the brain. OK107-GAL4 largely targeted expression to the mushroom body as previously described (Aso *et al.* , 2009). We next compared the expression levels driven by nSyb-GAL4, elav-GAL4 and OK107-GAL4 in the mushroom body ( Figure 1D ). Fluorescence intensity of mCD8::GFP was measured in each of the mushroom body lobes, and normalized to the intensity of the neural cell adhesion molecule fasciclin II (FasII), which is expressed strongly in the alpha and beta lobes, and weakly in the gamma lobes (Crittenden *et al.* , 1998) ( Figure 1E ). Negligible expression of mCD8::GFP was observed in the absence of GAL4, suggesting minimal leaky expression of this transgene. FasII intensity was consistent across all lobes and genotypes indicating that the presence of GAL4 or mCD8::GFP did not affect its expression. OK107-GAL4 drove the highest expression in all lobes compared to both elav-GAL4 and nSyb-GAL4, and there was no significant difference in lobe intensity between these two. It is important to note that the significant mCD8::GFP intensity at the tips of the alpha and gamma lobes observed in single sections of elav-GAL4>mCD8::GFP brains did not colocalize with FasII (due to the differential localization of these molecules) and therefore was not included for measurement, resulting in underestimation of elav-GAL4>mCD8::GFP expression compared to nSyb-GAL4>mCD8::GFP. Expression levels governed by OK107-GAL4 were also underestimated as mCD8::GFP was similarly abundant at the alpha lobe tips in OK107-GAL4>mCD8::GFP brains.

nSyb-GAL4 was selected for subsequent analysis of fine-tuning of expression with the AGES and TARGET systems. Repression of GAL80 with AGES is facilitated by fusion of GAL80 to an auxin-inducible degron (AID) tag that is ubiquitinated in the presence of auxin and targeted for degradation ( Figure 1F ) (McClure *et al.* , 2022). Fly media is supplemented with 1-naphthaleneacetic acid (K-NAA) auxin, which has been demonstrated to transverse the fly equivalent of the blood brain barrier, enabling de-repression in neuronal cells, with an effective concentration of 10 mM in adult flies (McClure *et al.* , 2022). The TARGET system utilizes a temperature-sensitive mutant of GAL80, such that when flies are raised at 19°C, GAL80 represses GAL4, but increasing temperature increases degradation of GAL80 and derepression of GAL4 (McGuire *et al.* , 2004).

We first compared the temporal profiles of induction between the systems. Flies were raised at 19°C and then transferred to 30°C to for induction of the TARGET system, or raised at 25°C and transferred onto 10 mM K-NAA food for induction of AGES. Flies were harvested periodically over a timecourse from 0 to 72 hours ( Figure 1G ). No mCD8::GFP was detected at the 0 hour incubation time point for AGES, however low levels were detected for the TARGET system, indicating incomplete repression of transgene expression ( Figure 1H ). This is consistent with a previous study that observed incomplete repression by GAL80ts at low temperatures with Mef2-GAL4 and DJ694-Gal4 throughout development and in the adult thorax (Barwell *et al.* , 2023). For both AGES and TARGET systems, expression increased over the 72 hour timecourse. The induced level of expression with the TARGET system was 15-fold, 14-fold and 7-fold higher than AGES at 24, 48 and 72 hours, respectively. We surmise that the difference in time taken to activate AGES may be a result of K-NAA having to cross the blood brain barrier, compared to the rate of conformational change achieved upon temperature change for TARGET.

We next measured the impact of dose of K-NAA on the induction profile of AGES in the brain. Following incubation for 24 hours on food containing K-NAA at 0, 5, 10, 20 and 40 mM, a positive linear correlation between concentration and level of mCD8::GFP expression was observed ( Figure 1I ). Treatment with 40 mM K-NAA resulted in a 2.6-fold increase in expression over 5 mM, however the expression mediated by 40 mM K-NAA was still more than two times lower than the leaky basal expression observed with the uninduced TARGET system, and five times less than that of TARGET flies raised at 30°C for 24 hours ( Figure 1J ).

These data demonstrate that elav-GAL4 and nSyb-GAL4, common neuronal drivers, have varying distribution in the adult brain, and promote a significantly lower level of expression in the mushroom body compared to OK107-GAL4. Both AGES and TARGET systems can be used for efficient and precise fine-tuning of the level of GAL4 activity in the adult brain. We found no evidence of leaky expression with AGES, which conferred stronger repression of GAL4 than the TARGET system. This suggests that AGES is an ideal platform for regulating the expression of toxic genes. However it should be noted that AGES does not achieve the same level of inducible expression as the TARGET system. Given the impact that auxin supplementation has on behavioral analyses (McClure *et al.* , 2022), and temperature has on the rate of development (Powsner, 1935) , lifespan (Mołoń *et al.* , 2020), and energy stores (Klepsatel *et al.* , 2019), these data provide insight into relative levels of control governed by each in neurons, which will aid in careful selection of the optimal system for specific experimental applications.

## Methods

**Fly strains**

All flies were raised on standard medium (per litre of dH _2_ O: 10 g agar, 40 g yeast, 110 g ground cornmeal, 20 mL molasses, 130 g sugar, 3.3 g methyl 4-hydroxybenzoate (Sigma Aldrich) dissolved in 37 mL 95% ethanol) on a 12 hour light/dark cycle. For induction of AGES, K-NAA (1-napthaleneacetic acid potassium salt, Phytotechnology Laboratories, cat no. N610) was diluted in water to 250 mM and added to freshly made fly food once the food had cooled to below 70°C, as described by McClure *et al.* (2022). Flies were raised at 25°C unless otherwise described in the figure legends for the TARGET system. Adult flies were transferred to medium containing the indicated concentrations of K-NAA to induce the AGES system, or 30°C to induce the TARGET system as indicated. Fly strains were obtained from the Bloomington *Drosophila* Stock Center as indicated in the reagents table.

**Immunohistochemical staining**

Whole flies were pre-fixed for one hour at room temperature in PFAT-DMSO (4% paraformaldehyde, 1x PBS, 0.1% Triton X-100, 5% DMSO) before dissection in 1x PBST (10 mM phosphate buffer, 2.7 mM potassium chloride and 0.137 M sodium chloride, 0.5% Triton X-100, pH 7.4), after which brains were post fixed for 20 minutes at room temperature in PFAT-DMSO and stored in 100% methanol at -20°C. Brains were rehydrated for 5 minutes in 50% methanol/PBST, washed 3 x 5 mins in PBST, blocked for three hours in 5% normal goat serum in PBST and incubated overnight at room temperature with anti-GFP (1:20,000) or anti-FasII (1:50) in 5% NGS/PBST. Following 3 x 5 minute washes in PBST brains were incubated overnight at 4°C with secondary antibody in 5% NGS/PBST. Brains were washed 3 x 20 minutes in PBST then mounted on a slide in Antifade (90% glycerol, 0.2% n-propyl gallate, 1x PBS) and stored at 4°C in the dark until imaging.

**Confocal microscopy imaging & intensity quantification**

Confocal microscopy was performed at the Manawatu Microscopy and Imaging Centre on a TCS SP5 DM6000B Confocal microscope. Optical sections were taken (1 μM through the whole brain and 2 μM through the mushroom body) and maximum projections of z-stacks were generated in ImageJ (Schneider *et al.* , 2012). For intensity quantification, lobes of the mushroom body (alpha, beta, and gamma) were traced on a single optical section (FasII channel) using the freehand ROI select and the Area, Mean, and IntDen recorded using the measure function. Background fluorescence was recorded in a region with no sample and used to calculate the corrected total lobe fluorescence (CTLF) as follows: CTLF = IntegratedDensity of lobe – (area of selected lobe x mean background fluorescence). CTLF was calculated for eight lobes per lobe type (alpha, beta, gamma) per genotype in both GFP and FasII channels. GFP CTLF was normalized to FasII CTLF.

**Western blotting**

Whole flies were collected in 15 mL tubes and snap frozen in a dry ice/ethanol bath before vortexing to snap heads from bodies. Heads were sorted from bodies over dry ice on a piece of acetate, homogenized in RIPA buffer (150 mM sodium chloride, 0.1% Triton X-100, 0.5% sodium deoxycholate, 0.1% SDS, 50 mM Tris, pH 8.0), centrifuged for two minutes at 13,000 g at 4°C, and the supernatant retained as the whole-cell lysate. Total protein was quantified using the BCA Protein Assay kit (ThermoFisher Scientific) as per manufacturer’s instructions and 30 µg of each sample was denatured in 1x Laemmli sample buffer (2% sodium dodecyl sulphate, 5% 2-mercaptoethanol, 10% glycerol, 0.01% bromophenol blue, 60 mM Tris HCl pH 6.8) at 95°C for five minutes before loading onto a 4-20% Mini Protean gel (BioRad) and electrophoresing at 200V in 1x running buffer (25 mM Tris, 190 mM glycine, 0.1% SDS). Protein was transferred onto a nitrocellulose membrane at 100V for one hour at 4°C in 1x transfer buffer (25 mM Tris, 190 mM glycine, 0.1% SDS, 20% methanol) which was then blocked at room temperature in 5% skim milk powder in TBST (20 mM Tris, 150 mM NaCl, pH 7.6, 0.1% Tween-20), washed 3 x 5 min in TBST then incubated overnight in primary antibody (anti-GFP, 1:4,000) in 1% milk powder/TBST. Following 3 x 5 min washes in TBST the membrane was incubated in secondary antibody in 1% milk powder/TBST for one hour at room temperature, washed 3 x 5 min in TBST and detected using ECL Prime (GE Healthcare) reagent and the Azure c600 Gel Imaging System (Azure Biosystems). Membranes were stripped in mild stripping buffer (0.2 M glycine, 3.5 mM SDS, 1% Tween, pH 2.2) for 2 x 10 min incubations at room temperature then washed 2 x 10 min in PBS (10 mM phosphate buffer, 2.7 mM potassium chloride and 0.137 M sodium chloride, pH 7.4) followed by 2 x 5 min washes in TBST before blocking and reprobing with anti-tubulin (1:500). Band intensity was quantified using ImageJ Gel Analyzer. Intensity values for mCD8::GFP were normalized to tubulin.

**Statistical analysis**

Statistical significance was assessed via Student’s t-test (n=2). Where n>2, statistical significance was assessed via one-way ANOVA followed by a post-hoc analysis with a Tukey’s HSD test. A post-hoc Tukey’s HSD test was only carried out if ANOVA provided a K value of more than two and analysis of variance gave a significant F-ratio. A p-value of less than 0.05 was considered to be statistically significant.

## Reagents

**Fly Strains**

**Table:** 

**Name**	**Genotype**	**Stock ID**
elav-GAL4	P{w[+mW.hs]=GawB}elav[C155]	BDSC 458
nSyb-GAL4	y[1] w[1118]; P{y[+t7.7] w[+mC]=nSyb-GAL4.P}attP2	BDSC 51941
OK107-GAL4	w[*]; P{w[+mW.hs]=GawB}OK107 ey[OK107]/In(4)ci[D], ci[D] pan[ciD] sv[spa-pol]	BDSC 854
Tubulin-GAL80ts (TARGET)	w[*]; P{w[+mC]=tubP-GAL80[ts]}10; TM2/TM6B, Tb[1]	BDSC 7108
Tubulin-TIR1-2A-GAL80-AID (AGES)	w[1118]; PBac{y[+mDint2] w[+mC]=tubP-TIR1-2A-GAL80.AID}VK00040	BDSC 92470
UAS-mCD8::GFP	y[1] w[*]; P{w[+mC]=UAS-mCD8::GFP.L}LL5, P{UAS-mCD8::GFP.L}2	BDSC 5137
nSyb-GAL4; UAS-mCD8::GFP	w[1118]; betaTub60D[Pin-1]/CyO; P{y[+t7.7] w[+mC]=nSyb-GAL4.P}attP2, P{w[+mC]=UAS-mCD8::GFP.L}LL6	BDSC 51944

BDSC: Bloomington *Drosophila* Stock Center

**Antibodies**

**Table:** 

**Name**	**Concentration**	**Catalogue number & source**
GFP	1:20,000 IHC 1:4,000 WB	Ab290, Abcam
FasII	1:50	1D4, DSHB
Alpha-tubulin	1:500	12G10, DSHB
Goat anti-Mouse Alexa 555	1:500	A-21422, Invitrogen
Goat anti-Rabbit Alexa 647	1:500	A-21244, Invitrogen
ECL Rabbit IgG HRP-linked	1:40,000	NA934VS, GE Healthcare
ECL Mouse IgG HRP-linked	1:20,000	NA931VS, GE Healthcare

IHC: immunohistochemistry, WB: western blotting, DSHB: Developmental Studies Hybridoma Bank.
